
JOURNAL INFORMATION
==============================
NLM Title Abbreviation: Med Chir Rev
NLM Journal ID: 0034617

The Medico-Chirurgical Review

Publisher: Biomedical Journal Digitization Project

ARTICLE INFORMATION
==============================
PMCID: PMC4966191
PMID: 29917613
Article version: 1
Subjects: Articles

Varieties

Print publication date: 1828 Jul 1
Volume: 9
Issue: 17
First page: 168
Last page: 168
1828 Apr 12
License: This work is free of known copyright restrictions. For more information, please see PMC Back Issue Digitization.
License URL: https://creativecommons.org/publicdomain/mark/1.0/

==============================

the consequences of this infernal system, unless some measure can be devised to check its ruthless career. The impression, however, is every day becoming more general, that the utter degradation of the medical character is at hand—and that medical men have been their own executioners in the encouragement which they have given to vituperative publications. The profession will learn wisdom in the school of adversity—but not till they are severely scourged for their folly.

### VARIETIES.

### 22. *Enlarged Livers and Spleens removed by Iodine.*

Three cases of this kind have recently been reported by Dr. Milligan, from the Royal Universal Infirmary for Children, which appear to prove the superiority of iodine over mercury in glandular and visceral tumours. In the first case, the child was five years of age, and presented a tumour occupying the right hypochondrium, extending anteriorly in a line with the linea alba, and inferiorly to the superior part of the hypogastric region. It was firm, colourless, pushed out the ribs considerably, and was attended occasionally with shooting pains. The tumour had been perceived growing for six months. The child was put on a course of the tincture of iodine, and in about two months and a half the tumour was quite gone.

In the second case, the child was under two years of age, and had an ague when admitted. There was a tumour in the region of the spleen, said to be of eight months growth. Mercury had been used without effect, and Dr. Milligan ordered the iodine in small doses. In little more than a month the swelling was gone. In the third case the tumour was lower than the region of the spleen; but it gave way to the tincture of iodine.

### 23. *Gangrene of the Lungs not fatal.*

Some pathologists of this country deny the possibility of recovery in cases where a portion of lung becomes disorganized in a gangrenous way, and where this disorganization is supposed to be indicated by the black fetid expectoration exhaling the odour or mortified parts. Laennec and others, on the Continent, maintain the possibility of such an occurrence, and we are inclined to side with such a man as Laennec, rather than with those who are opposed to him in this country. The following case was lately detailed by M. Laurent to the Parisian Academy of Medicine.

*Case.* Madam the Countes of L——, had experienced from youth several attacks of hæmoptysis. In a journey which she made to Versailles, in 1823, she was seized with acute pain in the right side of the chest, which yielded to the application of 24 leeches, but which still induced an indescribable malaise of two or three days' duration. On the 4th day, she was seized with a violent and convulsive cough, succeeded by an abundant expectoration of greenish matter mixed with a black substance, emitting a horrible gangrenous odour. This kind of expectoration continued a long time, and it was ten months before she perfectly recovered.—REVUE MED.

### 24. *Paralysis from Cubebs.*

Mr. Broughton has related a curious case of this kind, in the person of a young gentleman, who had been taking the above-mentioned medicine for about a fortnight, in the dose of two drachms thrice daily, for a gonorrhœa, and being otherwise in good health, and living quietly. A distortion of the mouth to one side, whenever he attempted to speak or smile, supervened—and the pulse became irregular, with some other symptoms of constitutional derangement. He was bled to half a pint, without any advantage. Then he was freely purged, with benefit, but at the end of three weeks the distortion had not entirely disappeared. Was this not an affection of the portio dura, and the result of cold caught in the side of the head, rather than the effect of cubebs?—MED. GAZ.

*N. B.* Fasciculus VII. which is printed by mistake at the top of page 1, should be Fasciculus I.
